# Morphine induces changes in the gut microbiome and metabolome in a morphine dependence model

**DOI:** 10.1038/s41598-018-21915-8

**Published:** 2018-02-26

**Authors:** Fuyuan Wang, Jingjing Meng, Li Zhang, Timothy Johnson, Chi Chen, Sabita Roy

**Affiliations:** 1 Department of Veterinary Population Medicine, University of Minnesota, 225 VMC 1365 Gortner Ave., St Paul, MN 55108 USA; 2 Department of Surgery and Sylvester Cancer Center, Miller School of Medicine, University of Miami, Miami, Florida 33101 USA; 3 Department of Pharmacology, University of Minnesota, 515 Delaware St SE, Moos 11-204, Minneapolis, MN 55455 USA; 4 Department of Veterinary and Biomedical Sciences, University of Minnesota, 225 VMC 1365 Gortner Ave., St Paul, MN 55108 USA; 5 Department of Food Science and Nutrition, University of Minnesota, 1334 Eckles Ave, St Paul, MN 55108 USA

## Abstract

Opioid analgesics are frequently prescribed in the United States and worldwide. However, serious comorbidities, such as dependence, tolerance, immunosuppression and gastrointestinal disorders limit their long-term use. In the current study, a morphine-murine model was used to investigate the role of the gut microbiome and metabolome as a potential mechanism contributing to the negative consequences associated with opioid use. Results reveal a significant shift in the gut microbiome and metabolome within one day following morphine treatment compared to that observed after placebo. Morphine-induced gut microbial dysbiosis exhibited distinct characteristic signatures, including significant increase in communities associated with pathogenic function, decrease in communities associated with stress tolerance and significant impairment in bile acids and morphine-3-glucuronide/morphine biotransformation in the gut. Moreover, expansion of *Enterococcus faecalis* was strongly correlated with gut dysbiosis following morphine treatment, and alterations in deoxycholic acid (DCA) and phosphatidylethanolamines (PEs) were associated with opioid-induced metabolomic changes. Collectively, these results indicate that morphine induced distinct alterations in the gut microbiome and metabolome, contributing to negative consequences associated with opioid use. Therapeutics directed at maintaining microbiome homeostasis during opioid use may reduce the comorbidities associated with opioid use for pain management.

## Introduction

Morphine is the gold standard for pain management. Opioid analgesics are frequently prescribed in the United States and worldwide^1^. However, serious side effects, such as addiction, analgesic tolerance, immunosuppression and gastrointestinal (GI) symptoms limit their use^1–3^. Peripheral consequences of opioid use, such as constipation, nausea, vomiting, bloating and gut barrier dysfunction have been well-documented^4^. More recently, alteration in the gut microbiome has been implicated in opioid-induced bowel dysfunction and gut barrier disruption^5^. However, the mechanisms underlying morphine-induced dysbiosis are still unknown. The recent rapid progress in metagenomics has provided powerful tools to determine if perturbation of the human microbiome contributes towards disease^6^. Changes in the composition or density of the microbiota are associated with increased susceptibility to a variety of pathogens and abnormal mucosal immune responses^7,8^. Taxonomic and functional diversity of gut microbiota is crucial in conferring resilience in gut homeostasis^9^. Low microbial diversity correlates with obesity^10,11^, inflammatory bowel disease (IBD)^12^, and recurrent *Clostridium difficile*-associated diarrhea (CDAD)^13^. It is still unclear if there is a direct cause-consequence relationship between microbial diversity and resilience. Consistent with other reports, we have demonstrated that morphine disrupts intestinal barrier function and induces bacterial translocation in mice^14,15^. Use of opioids is associated with an increased risk of *C. difficile* infection^16^. We recently demonstrated that morphine inhibition of endotoxin tolerance, leading to sustained sepsis, is mediated by modulation of miR-146a^17^, and opioid exacerbation of gram-positive sepsis is rescued by IL-17A neutralization^15^. Our recent study demonstrated that opioid-induced gut microbial disruption and bile acid dysregulation leads to gut barrier compromise and sustained systemic inflammation^18^. It is not yet clear whether morphine treatment perturbs gut microbial homeostasis, resulting in increased growth of potential pathogenic bacteria in the gut. Several studies are cross-sectional and do not have the ability to examine changes over time with repeated biosample measurements. To date, there are no reports examining the association of opioid treatment with dynamic changes in the microbiome and metabolome in a short-term study.

There is evidence suggesting that metabolites in the gut play a significant role in the crosstalk between gut microbes and host biological functions, such as maturation of the host immune system^19^ and protection against pathogens^20^. Disruption in bile acid metabolism is associated with increased susceptibility to *C. difficile* infection^21^. Gastrointestinal barrier function may be regulated by intestinal symbiotic bacterial metabolites through xenobiotic sensor PXR-dependent TLR4 signaling^22^. Healthy microbiota produce short chain fatty acids (SCFAs) as carbon sources for the host, synthesize vitamins and essential amino acids, transform bile acids, produce neurotransmitters and modify xenobiotics^23^. Metabolomic analysis allow for the determination and identification of small molecular metabolites within the gut lumen, thus profiling the functional status of the gut microbiome^24^. We have confirmed that morphine-induced intestinal barrier dysfunction contributes to bacterial translocation^14^. However, it is still unknown how morphine treatment modulates the composition and abundance of gut metabolites. The current study, therefore, is focused on identification of distinctness in the morphine-modulated gut microbiome and its functional consequences through metabolomic analysis.

The host-microbiome metabolic interaction affects xenobiotic metabolism significantly^25^. Clayton, *et al*., demonstrated that the host-gut microbial metabolic interaction leads to modifications of major xenobiotic-metabolizing cytochrome enzymes and alteration of bile acid metabolites^25,26^. Morphine is metabolized primarily through glucuronidation, biotransforming to morphine 3-glucuronide (M3G) and morphine 6-glucuronide (M6G) in the liver^27^. Though M3G exhibits no analgesic effect, M6G is more potent than morphine^28^. M6G and M3G are hydrolyzed by β-glucuronidase, synthesized by both intestinal mucosal cells and gut bacteria, and subsequently reabsorbed as morphine^29–31^. Our recent study demonstrated that glucocorticoids can significantly augment morphine-withdrawal-induced immunomodulation^32^. However, the role of the gut microbiome in morphine metabolism and elimination is relatively unknown.

*The* *aim of the present study is to evaluate the effect of morphine use or abuse on the temporal shift in the gut microbiome and its consequence on the metabolome in a short-term study*. The composition of the gut microbiome and its metabolomic functions were determined at day 0, day 1, day 2, day 3, day 4, day 5 and day 6 following morphine treatment. Our results demonstrate that morphine treatment induces a decrease in the diversity of the microbial community and leads to distinct clustering and profiling of the gut microbiome and metabolome when compared to the observations in placebo-treated mice. We hypothesized that expansion of *Enterococcus faecalis* is a distinct feature associated with opioid-induced gut microbiome alteration and alterations in deoxycholic acid (DCA) and phosphatidylethanolamines (PE) are associated with opioid-induced metabolomic changes. We demonstrated that *E. faecalis* augmented tolerance of morphine analgesic effect in mice. Moreover, we determined an increase in the morphine-3-glucuronide/morphine (M3G/MS) ratio in the gut, suggesting reduced conversion of M3G by deconjugating microbes to morphine. These results suggest that morphine-induced changes in the gut microbiome have implications in the enterohepatic recirculation of morphine and, thereby, its efficacy as an analgesic agent. In addition, we demonstrate a cross-correlation and an association between reduced intestinal bacterial communities and bile acid metabolism. Collectively, these results demonstrate that morphine induces a distinct alteration in the gut microbiome and metabolome contributing to opioid-induced pathogenesis and disrupted morphine pharmacokinetics.

## Results

### Morphine treatment leads to temporal modulation of the gut microbiome

**Figure 1:**
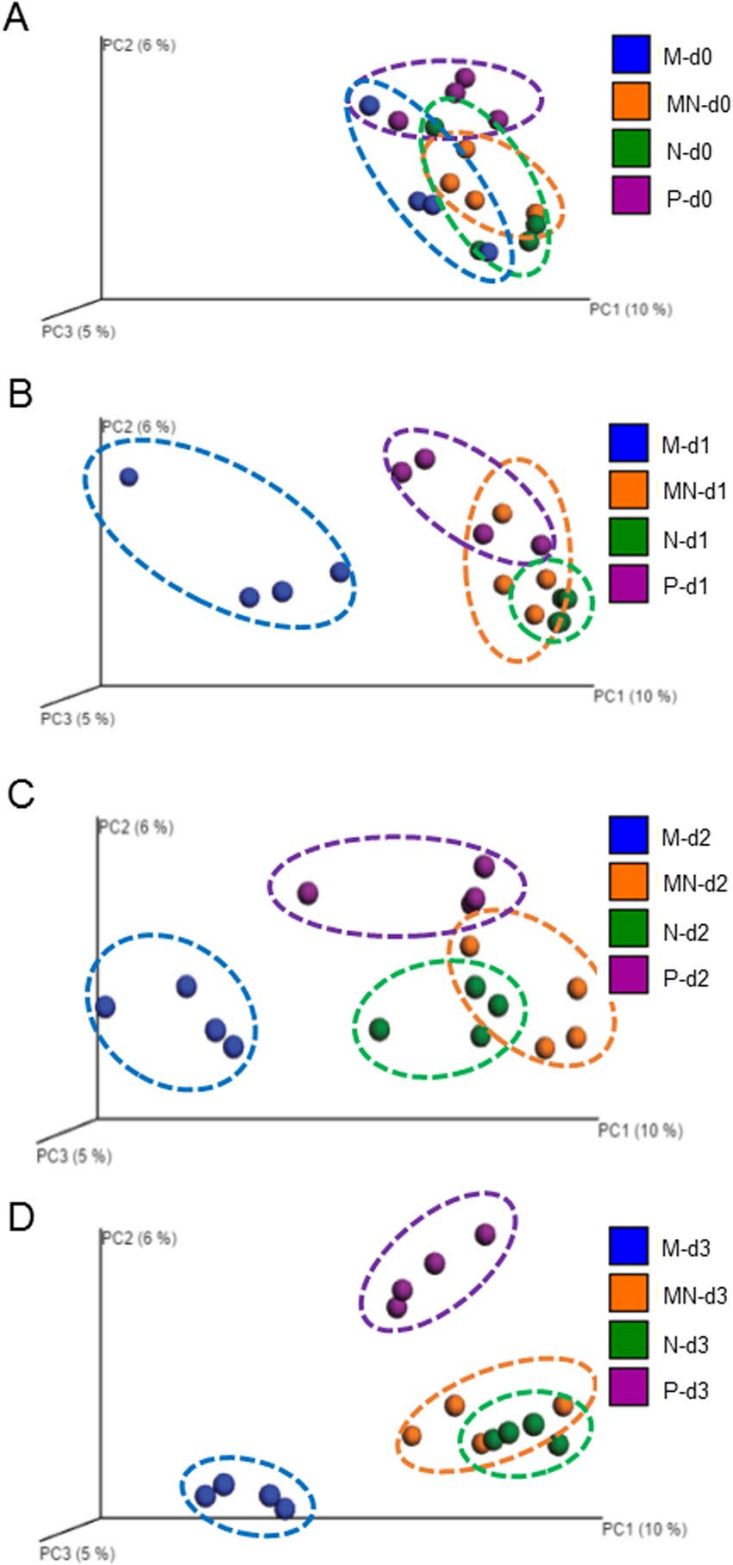
Morphine treatment leads to temporal modulation of the gut microbiome in 3 days post-treatment. Beta diversity measures of the gut microbiome following treatment with placebo, morphine, naltrexone, or morphine plus naltrexone. Wild-type mice were implanted with placebo (P), 25 mg morphine (M), 30 mg naltrexone (N), or morphine and naltrexone (MN) pellets subcutaneously (n = 4 in each group). Fecal samples were taken for analysis at the following time points: day 0, day 1, day 2, and day 3 post-treatment. Principal coordinates analysis of samples from day 0 (**A**), day 1 (**B**), day 2 (**C**) and day 3 (**D**) using the UniFrac metric at the OTU level. In Fig. 1A, F value = 4.766, total degree of freedom (DF) = 27, DF(treatment, between columns) = 2, DF(residual, within columns) = 25. In Fig. 1B, F = 1.789, DF (total) = 27, DF(treatment, between columns) = 2, DF(residual, within columns) = 25. In Fig. 1C, F = 17.25, DF (total) = 27, DF(treatment, between columns) = 2, DF(residual, within columns) = 25. In Fig. 1D, F = 89.39, DF (total) = 27, DF(treatment, between columns) = 2, DF(residual, within columns) = 25. Mantel test is run over these distance classes versus the ecological distance matrix. Parametric p-value (Bonferroni-corrected) < 0.01.

**Figure 2:**
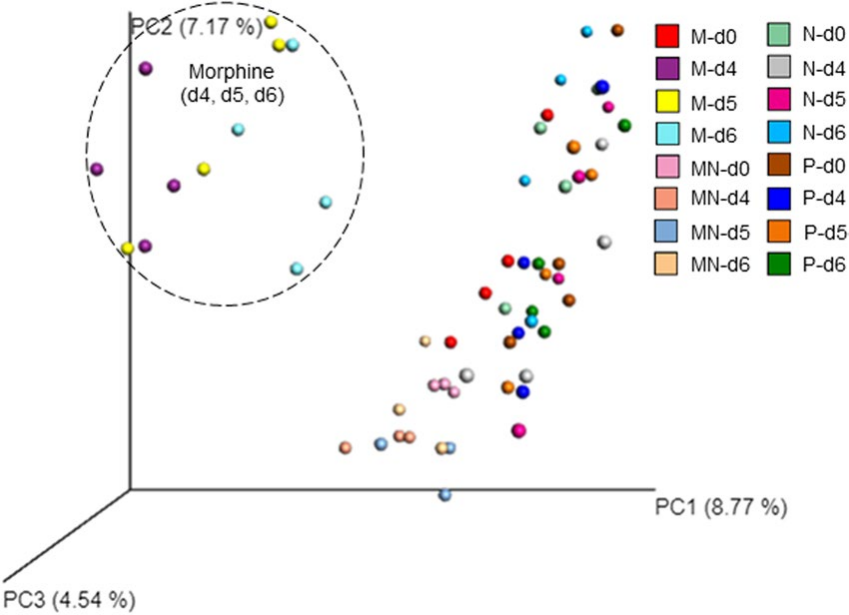
Morphine treatment leads to sustained modulation of the gut microbiome for 6 days post-treatment. Beta diversity measures of the gut microbiome following treatment with placebo, morphine, naltrexone, or morphine plus naltrexone. Wild-type mice were implanted with placebo (P), 25 mg morphine (M), 30 mg naltrexone (N), or morphine and naltrexone (MN) pellets subcutaneously (n = 4 in each group). Fecal samples were taken for analysis at the following time points: day 0, day 4, day 5, and day 6 post-treatment. Principal coordinates analysis of samples from day 0, day 4, day 5, and day 6 post-treatment using the UniFrac metric at the OTU level. F value = 7.304. Total degree of freedom (DF) is 495, including DF of treatment (between columns), which is 35, and DF of residuals (within columns), which is 460. A Mantel test is run over these distance classes versus the ecological distance matrix. Parametric p-value (Bonferroni-corrected) < 0.01.

Morphine treatment has been demonstrated, in our laboratory and also in human patients, to induce bacterial translocation on day 2 post-treatment^2,14,33^. It has been demonstrated that microbial dysbiosis contributes to bowel dysfunction and susceptibility to infectious diseases^5,34^. To determine the effect of morphine treatment on the gut microbial profile, we analyzed the gut microbiome based on Illumina sequencing of intestinal microbial 16S-rRNA genes. Interestingly, opioid-induced adverse effects, such as constipation, are observed in patients within 1 day following morphine administration^35^. At day 1 post-treatment, mice receiving morphine revealed bacterial translocation into the mesenteric lymph node (MLN) and liver^14^. To determine time-dependent changes in the microbial composition following subcutaneous morphine pellet implantation, fecal samples were collected from the same animal at day 0, day 1, day 2 and day 3 following morphine treatment. Control animals were implanted subcutaneously with a placebo pellet. Our time course study revealed distinct modulation of the gut microbiome by morphine (Fig. 1). Principal coordinate analysis of fecal samples from day 0, day 1, day 2, and day 3 post-morphine treatment demonstrated that the microbial profiles at day 0 in all treatment groups were similar and there was no distinct clustering. However, as early as day 1, the microbiome from the morphine treatment group clustered distinctly from all other groups and the clustering was more pronounced, with all samples clustering more tightly, at day 3 post-morphine treatment. We expanded the study to 6 days post-treatment (Fig. 2). Data from day 4, day 5 and day 6 revealed the same trend as that of the 3-day experiment.

To determine the role of opioid receptors in morphine-induced effects, naltrexone–an opioid receptor antagonist–was implanted subcutaneously in mice to determine whether naltrexone antagonizes morphine-induced shifts in the gut microbiome. Results demonstrated that naltrexone antagonized morphine-induced alterations of the gut microbiome (Figs 1 and 2). Interestingly, animals treated with naltrexone alone clustered distinctly from the placebo group at day 3 following implantation, suggesting that endogenous opioids may set a basal tone on the host microbial profile (Fig. 1D).

**Figure 3:**
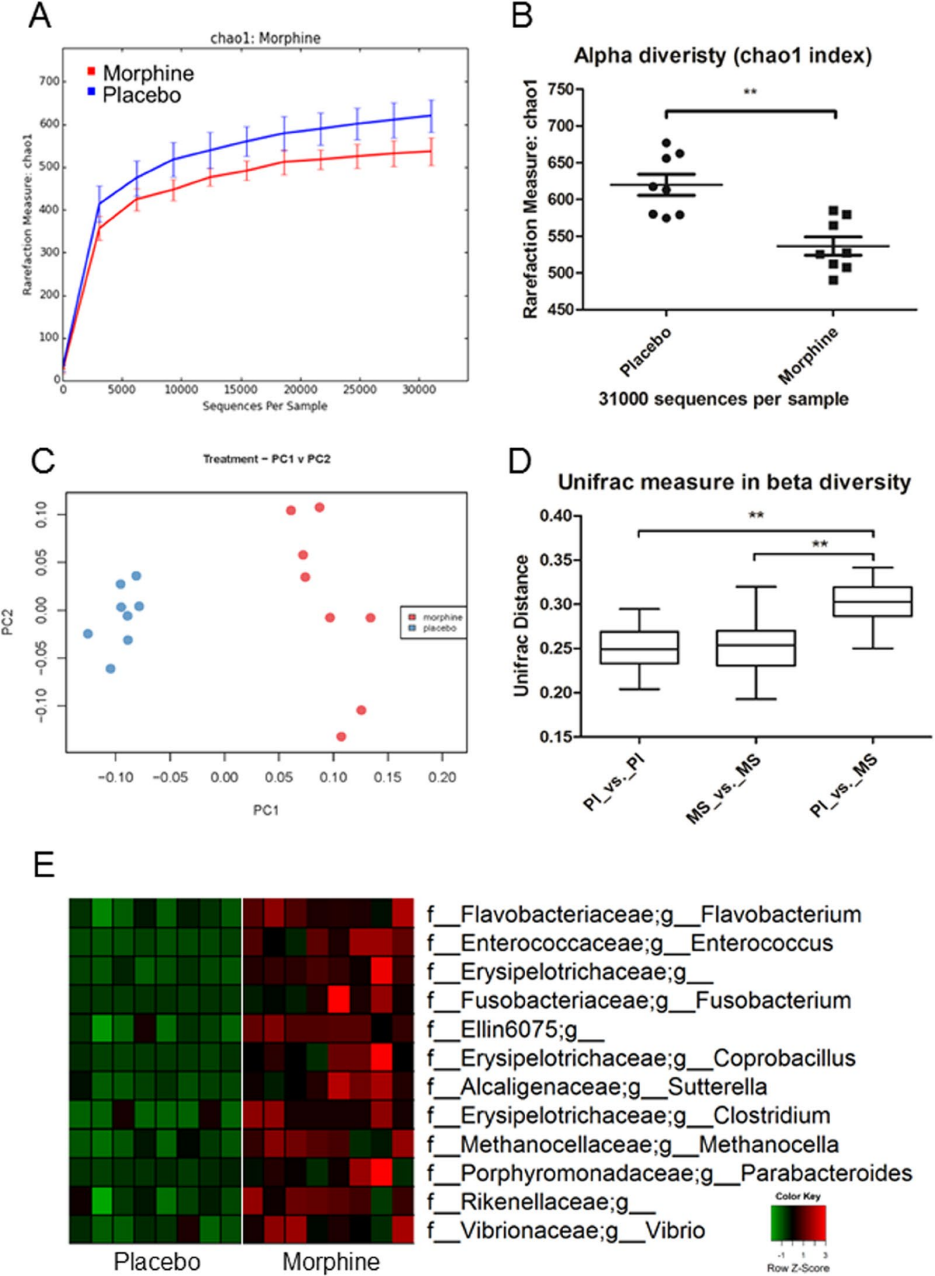
Morphine treatment induces distinct changes in the gut microbiome. Wild-type mice were implanted with placebo or 25 mg morphine pellets subcutaneously. Fecal samples were taken for analysis at day 3 post-treatment. (**A**) Alpha diversity was assessed using the chao1 index. Morphine treatment (n = 8) results in decreased alpha diversity compared to that of controls (n = 8) measured by using the chao1 index. The OTU tables were rarefied at the cutoff value of 31000 sequences per sample. (**B**) t-test was conducted on the chao1 index. **Indicates a significant difference, *P* value = 0.0030. (**C**) Principal coordinates analysis (PCoA) of samples using the UniFrac metric at the OTU level. (**D**) UniFrac distance significant tests were performed using QIIME. The tests of significance were performed using a two-sided student’s t-test. *Parametric p-value (Bonferroni-corrected) < 0.05, **Parametric p-value (Bonferroni-corrected) < 0.01. ANOVA test indicated P < 0.0001, F value = 63.29. Total degree of freedom (DF) is 119, including DF of treatment (between columns), which is 2 and DF of residuals (within columns), which is 117. (**E**) Morphine treatment results in a significant increase in pathogenic bacteria. Multiple hypothesis test with the given threshold (FDR = 0.05) demonstrates that the relative abundance of potential pathogenic bacteria (genus level) increases significantly at day 3 post-treatment with morphine compared to that following placebo treatment. Increased (red color) representative pathogenic bacteria include *Flavobacterium, Enterococcus, Fusobacterium, Sutterella, Clostridium*.

To compare microbial patterns, principal coordinate analysis (PCoA) was used. Principal coordinate analysis (PCoA = multidimensional scaling, MDS) is a method to explore and to visualize inter-object similarity/dissimilarity in a low-dimensional, Euclidean space. PCoA of unweighted UniFrac phylogenetic distances between microbial communities were carried out using this program with observation ID level. A 3D plot, based on unweighted UniFrac distance matrices obtained from the sequences at OTU level with 97% similarity, demonstrated a distinct clustering of the community composition between the morphine- and placebo-treated groups. By using the chao1 index to evaluate alpha diversity (diversity within a group) and using unweighted UniFrac distance to evaluate beta diversity (diversity between groups, comparing microbial community based on compositional structures), we observed that morphine treatment resulted in a significant decrease in alpha diversity (Fig. 3A,B) and shift in fecal microbiome at day 3 post-treatment compared to that after placebo treatment (Fig. 3C,D). Taxonomical analysis demonstrated that morphine treatment resulted in a significant increase in the potential pathogenic bacterial community. Multiple hypothesis test with the given threshold of an estimate of false discovery rate (q-value < 0.05) demonstrated that the relative abundance of potential pathogenic bacteria (genus level) increased significantly at day 3 post-treatment with morphine compared to that after placebo treatment (Fig. 3E and Supplementary Table S1). Representatives of potential pathogenic bacteria with an increased abundance post-morphine treatment at the genus level, include *Flavobacterium, Enterococcus, Fusobacterium, Sutterella, Clostridium*.

### Identification of association between gut microbial dysbiosis and morphine treatment at the species level

**Figure 4:**
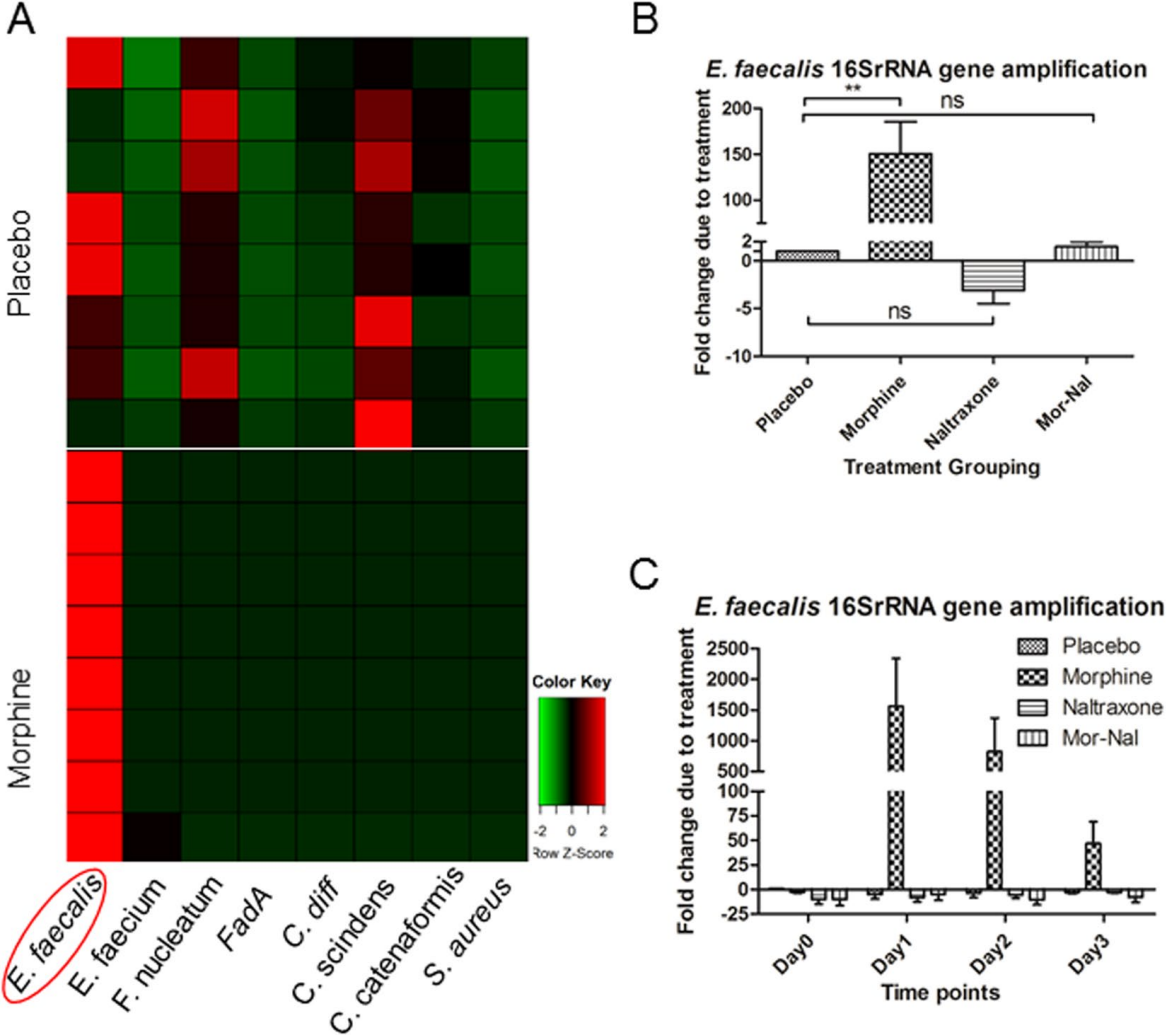
*Enterococcus faecalis* is a biomarker of morphine-induced alteration of the gut microbiome. Real-time PCR expression profiling of species-specific 16S-rRNA gene in gut microbiota. (**A**) The expression of species-specific 16S-rRNA gene was profiled in stool samples from mice *s.c*. implanted with placebo (control) or morphine using a qPCR assay (n = 8 in each group). The heat map was generated by a log transformation of the real-time PCR data presented as ΔCT (CT_species – CT_universal_16SrRNA)^60^. Red color indicates increased levels of amplification. (**B**) *E. Faecalis* 16S-rRNA gene amplification fold change due to treatments on day 3 post-treatment (n = 4 in each group). (**C**) *E. Faecalis* 16S rRNA genes amplification fold change due to treatments in a short-term study (n = 8 in each group).

To identify association between microbiome alteration and morphine treatment at the species level, we conducted expression profiling of selected species-specific 16SrRNA genes in the gut microbiota using quantitative real-time PCR. Our results demonstrate a significant expansion in the *Enterococcus faecalis* species in the morphine-treated group (Fig. 4A) with *E. faecalis* 16S-rRNA gene amplification 100 times greater in the morphine-treated group compared to the levels in the placebo-treated group on day 3 post-treatment (Fig. 4B). The effect of morphine on *E. faecalis* expansion was observed as early as day 1 in morphine-treated animals and sustained through all days tested post-treatment (Fig. 4C). The effect of morphine on *E. faecalis* abundance in the gut microbiome was antagonized by naltrexone treatment (Fig. 4B,C).

**Figure 5:**
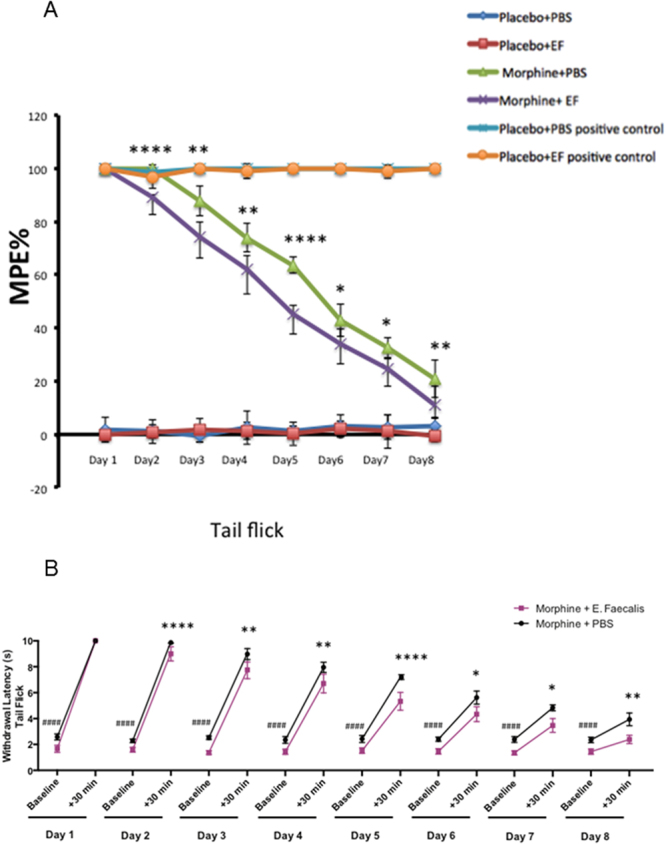
*Enterococcus faecalis* infection accelerates morphine induced analgesic tolerance. The mice were administrated with 5 g/L of streptomycin sulphate in the drinking water for 2 days and switch to normal drinking water for 24 hours before *E. faecalis* (EF) inoculation by oral gavage. The spectinomycin sulphate selective *E. faecalis* were diluted up to the concentration of 2 × 10^10^/mL in phosphate buffered saline (PBS). Each mouse was administered with 200ul spectinomycin solution by oral gavage daily. After 48 hours post gavage, the mice were treated with 250 mg/L spectinomycin sulfate (to prevent overgrowth of pathogenic gram-negative bacteria) in the drinking water during the behavior study. To maintain the population of *E. faecalis* in the mouse gut, mice were administered the same dose of *E. faecalis* and the same dose of spectinomycin sulphate by oral gavage daily during the behavior experiment for 8 days. (**A**) Analgesic effectiveness of morphine was evaluated by mouse reaction to heat. Analgesic tolerance, interpreted as percentage of maximum possible effect (MPE%), was determined by tail flick analgesic test. Mice were intraperitoneally injected with saline or 15 mg/kg morphine twice daily for 8 days with 12 hours interval. Behavioral assessment was performed before and 30 min after saline or morphine administration in the morning. All groups had a minimum of 10 mice/group. For positive controls, a new group of 10 mice treated with Placebo + PBS and a new group of 10 mice treated with Placebo + *E. faecalis* were administered with 15 mg/kg morphine at each time point to test analgesic reaction of these naive mice to morphine treatment. T-test analyses were used to compare MPE% in each group daily. P value of 0.05 or less was considered significant. (**B**) Daily nociceptive behavior and morphine antinociception throughout an 8-day chronic morphine schedule (15 mg/kg, intraperitoneally, twice daily): antinociceptive behavior by tail flick. Voltage to the light source was adjusted to achieve baseline latency between 2–3 seconds. The cut-off time is 10 seconds to avoid tissue damage. The mice were put on the tail flick assay for 5 min of habituation everyday for two days before the behavior test. The averages of each two measurements before and after morphine injection were recorded as baseline and response to morphine antinocipective effect at each time point daily during experimental period. The tests of significance were performed using a two-sided student’s t-test. *: morphine compared to morphine + *E. faecalis* 30 min after morphine injection, #: morphine compared to morphine + *E. faecalis* baseline. *p < 0.05, **p < 0.01 and ***p < 0.001, #### and ****p < 0.0001.

We next determined if infection with *E. faecalis* accelerates morphine analgesic tolerance in a tail flick test. Withdrawal latencies of the tail from a radiant heat source were measured by tail flick. We demonstrate that infection with *E. faecalis* augmented morphine analgesic tolerance compared to mice treated with morphine alone (Fig. 5A,B). Mice, who received repeated dose of morphine, presented 50% MPE% between day 5 and day 6 in the tail flick test. However, tolerance to morphine induced analgesia was observed in mice infected with *E. faecalis* in the presence of repeated morphine injections at day 2 compared to non-infected mice which developed tolerance at day 4 post morphine injection. The 50% MPE% occurred between day 4 and day 5 in mice infected with *E. faecalis*, who achieved 50% MPE% one day earlier than mice given PBS. Interestingly, the baseline withdrawal latency in mice infected with *E. faecalis* was lower than that of mice treated with PBS indicating that mice infected with *E. faecalis* were more sensitive to pain than uninfected mice. Based on these results, we propose that expansion of *E. faecalis* can be used as a potential biomarker of gut dysbiosis following morphine treatment.

### Morphine changes gut metabolomic profile gradually and shifts metabolites differentially

**Figure 6:**
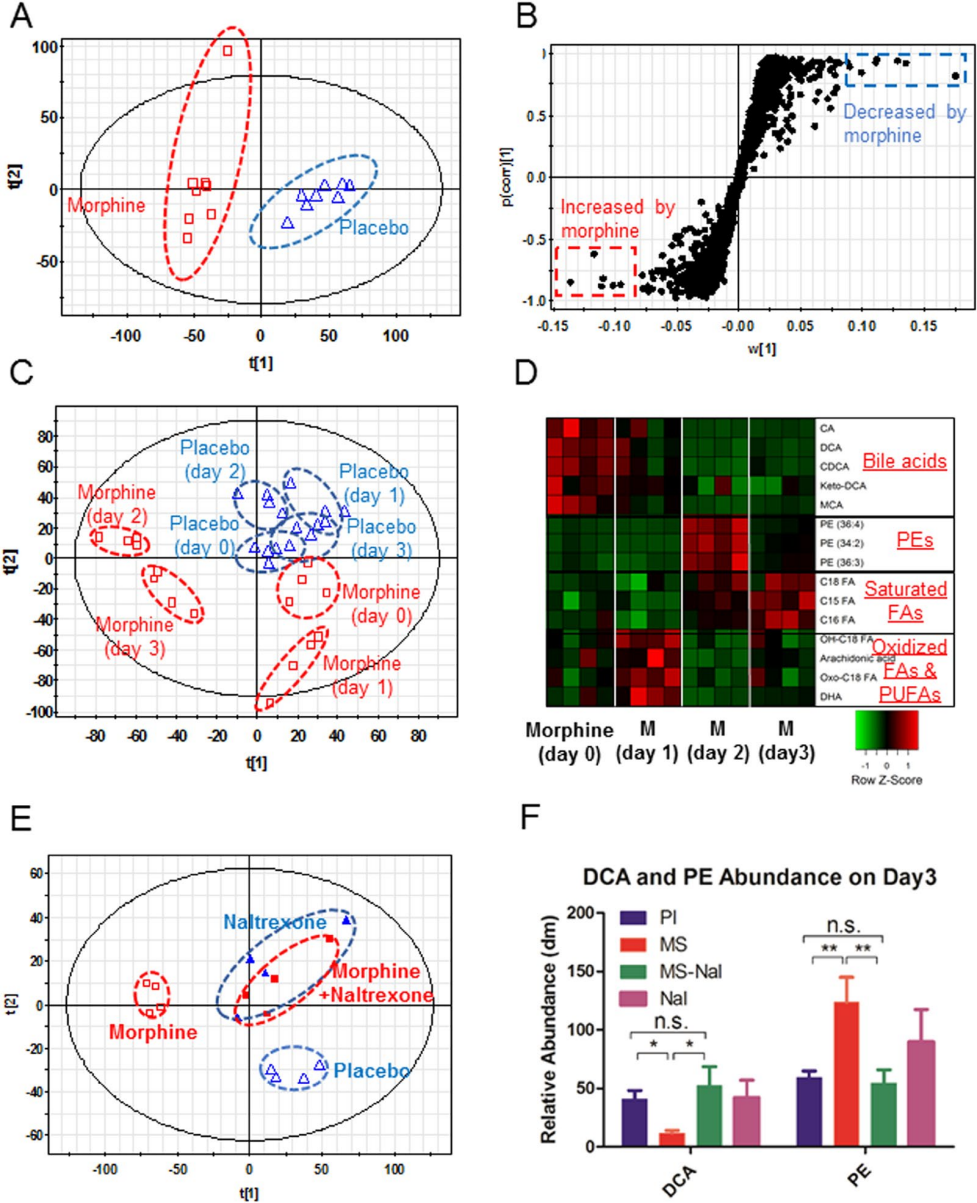
Metabolomic analysis of fecal matter and identification of significant shifts of gut metabolome following morphine treatment. (**A**) Mice were treated with 25 mg morphine or placebo pellet subcutaneously (n = 8 in each group). Fecal samples were taken for analysis at 3 days post-treatment. Scores scatter plot of the partial least square discriminant analysis (PLS-DA) model of fecal samples from wild-type mice (C57B6/J) with morphine (□) or placebo (Δ) treatment. The t[1] and t[2] values represent scores of each sample in principal components 1 and 2, respectively. (**B**) Loadings plot of the principal components (n = 8 in each group). Metabolites contributing to the differences in fecal samples from mice following morphine and placebo treatment were labeled, and their chemical identities were confirmed. (**C**) In a short-term study, fecal samples were taken from mice at the following time points: day 0, day 1, day 2, and day 3 post-morphine. Scores scatter plot of the partial least square discriminant analysis (PLS-DA) model of fecal samples from wild-type mice (C57B6/J) with morphine (□) or placebo (Δ) treatment at day 0, day 1, day 2, and day 3 post-treatment (n = 4 in each group). The t[1] and t[2] values represent scores of each sample in principal components 1 and 2, respectively. (**D**) Heat map plot of significant associations with morphine treatments and the loading of indicator metabolites in fecal matter from mice at day 0, day 1, day 2, and day 3 post-treatment (n = 4 in each group). All relative abundances are row z-score-normalized for visualization. (**E**) Metabolomic analysis of fecal samples and measurement of effects of naltrexone on morphine-induced gut metabolomic shifts at day 3 post-treatments. Mice were treated with placebo, 25 mg morphine, 30 mg naltrexone, or morphine + naltrexone pellets subcutaneously. (**F**) Relative abundance analysis of metabolites reveals naltrexone–an opioid receptor antagonist–reversed the effect of morphine on loading of deoxycholic acid (DCA), a secondary bile acid, and phosphatidylethanolamines (PEs), a class of phospholipids found in biological membranes. The tests of significance were performed using a two-sided student’s t-test (n = 4 in each group at day 3 post-treatments).

Microbial dysbiosis leading to disruption in host-microbe homeostasis does not only result in alteration of the microbial composition, but, more importantly, also disrupts the functional configuration of the microbiota^36^. Intestinal microbiota metabolizes input substrates from the host, including diet and xenobiotics, into metabolites that may affect the host^37^. Although gut microbiome may vary in different individuals, the core functional metabolic interaction with gut microbiota is essential for the host and alteration of the core functional microbiome is associated with different physiological states^38^. Thus, the identification of gut metabolomic profile is fundamental to identifying the functional changes in the gut microbiome. To explore changes in the metabolomic profile induced by morphine treatment, a time course study was carried out, as described earlier. Fecal samples were collected from the same animal at day 0, day 1, day 2 and day 3 post-treatment and were analyzed using liquid chromatography-mass spectrometry (LC-MS). Gut metabolomic analysis was performed on the same fecal samples as those that were used for microbial analysis. Scores scatter plots of the partial least square discriminant analysis (PLS-DA) model of fecal samples were constructed for morphine- or placebo-treated samples (Fig. 6A). The results demonstrated that morphine treatment induced a distinct gut metabolomic profile compared to that observed in the placebo-treated group.

To identify metabolites contributing to differences in fecal samples from mice following morphine or placebo treatments, we constructed a loading plot of the principal components in which each dot represents a single molecular metabolite (Fig. 6B). The metabolic profiles following morphine treatment were determined in a time course study. Our results demonstrate that morphine treatment resulted in a gradual and differential shift in metabolites in a time-dependent manner (Fig. 6C,D). To identify shifts in the gut metabolome following morphine treatment, we constructed scored scatter plots of the partial least square discriminant analysis (PLS-DA) model and characterized the metabolomics profiles of fecal samples from wild-type mice (C57B6/J) with those of morphine- or placebo-treated mice at day 0, day 1, day 2, and day 3 following treatment (Fig. 6C). Morphine treatment revealed a distinct clustering in the metabolomics profile compared to that observed after placebo treatment at each time point tested.

To reveal the details of the metabolomic changes due to morphine treatment, we identified the chemical increases and decreases following morphine or placebo treatment (Fig. 6D). The results demonstrate that morphine treatment altered the gut metabolomic profile and shifted metabolites differentially in the fecal samples taken from mice at day 1, day 2, and day 3 post-morphine treatment compared to that observed after placebo treatment. Bile acids decreased, whereas phosphatidylethanolamines (PEs) and saturated fatty acids increased as a consequence of morphine treatment (Fig. 6D).

### Naltrexone antagonized the morphine-induced gut metabolomic shift and reversed effects of morphine on bile acid metabolism

We next used an opioid receptor antagonist, naltrexone, to determine the role of classical opioid receptors in the morphine-induced effects on the gut metabolomics profile. Naloxone treatment completely antagonized the effects of morphine on gut metabolomics and reversed the effect of morphine on bile acid metabolism (Fig. 6E,F). Particularly, we established that the abundance of the secondary bile acid, deoxycholic acid, significantly decreased following morphine treatment and the morphine-induced decrease was antagonized by naltrexone (Fig. 6E,F). The abundance of the phospholipid, PE–a major component of the cell membrane–was increased by morphine treatment, indicating increased cell injury, and the morphine-induced increase was reversed by naltrexone treatment (Fig. 6F). These results indicate that levels of deoxycholic acid (DCA) and phosphatidylethanolamines (PE) may be used as biomarkers to indicate morphine-modulation of the gut microbiome and metabolome.

### Morphine-induced dysbiosis disrupts morphine metabolism and its enterohepatic recirculation

**Figure 7:**
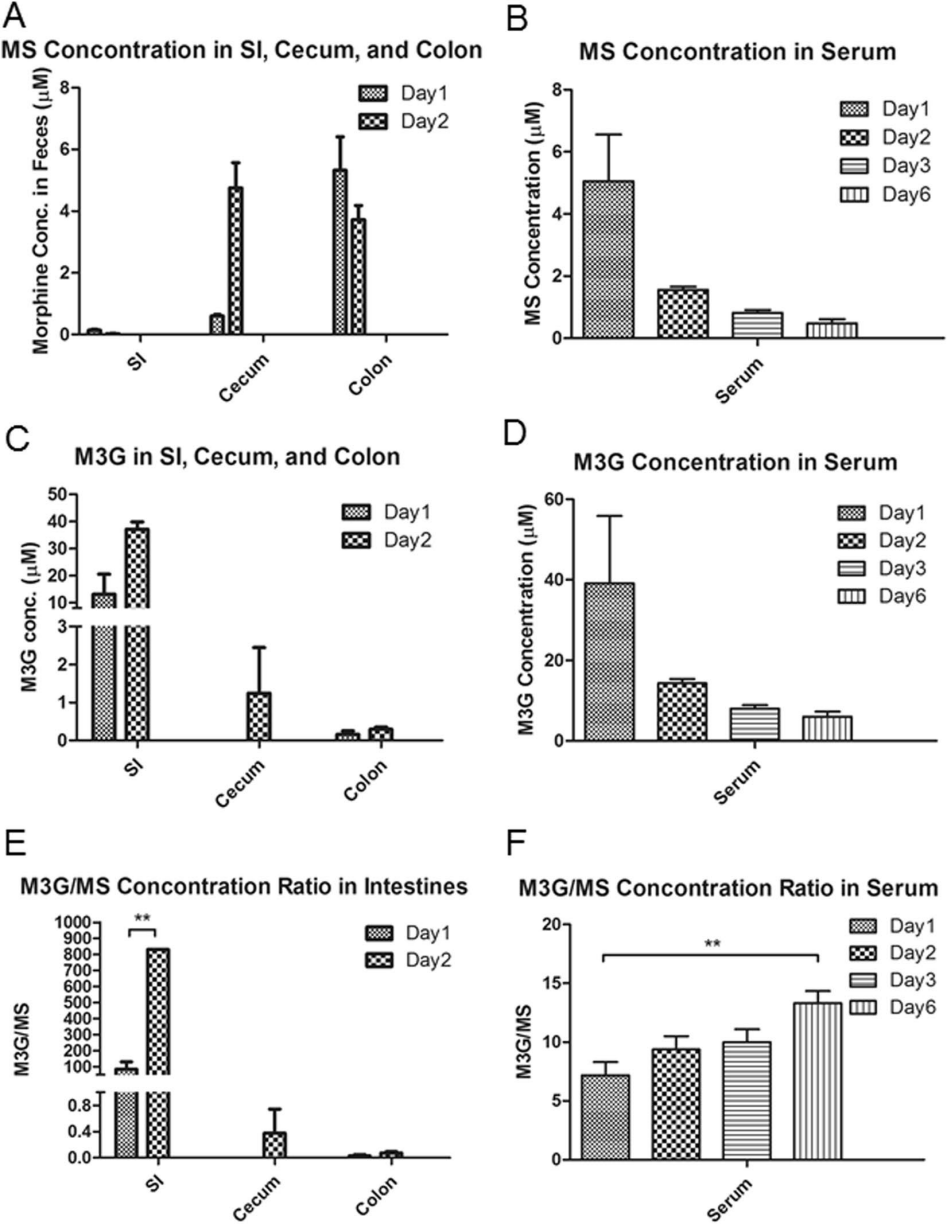
The M3G/MS concentration ratio in serum and fecal matter increases post-morphine treatment. LC-MS analysis identified MS and M3G concentrations. The M3G/MS concentration ratio increases following morphine treatment in both mouse serum and intestinal matter. Statistical significance tests were performed using a two-sided student’s t test (P < 0.05).

Morphine is conjugated to morphine-3-glucuronide (M3G) and morphine-6-glucuronide (M6G) in the liver and excreted into the gut through the biliary tract. M3G is totally inactive, whereas M6G appears to display a stronger analgesic activity than morphine^39^. The polar nature of the glucuronides prevents their reabsorption across the gut lumen. Intestinal de-conjugating bacteria transform M3G and M6G to morphine, which allows its reabsorption back into systemic circulation^40^. The enterohepatic circulation plays an important role in morphine elimination, which is characterized by a prolonged terminal elimination phase^41^. In mice and rats, M6G formation is insignificant; therefore, morphine is eliminated significantly as M3G. We demonstrated that the ratio of M3G/MS concentration in the intestinal lumen increased between day 1 and day 2 post-treatment (Fig. 7E) and serum levels also increased between day 1 and day 6 post-morphine treatment, indicating decreased M3G conversion to morphine by deconjugation in the gut (Fig. 7F).

To further clarify whether morphine-modulated gut microbiome alteration is associated with gut metabolomic changes following morphine treatment, we performed cross-correlation analyses between the gut microbiome and the metabolome (Supplementary Figure S1). The cross-correlation demonstrated that cholic acid and octadecanedioic acid were negatively associated with *Enterococcus* and *Erysipelotrichaceae* at the family level. Phosphatidylethanolamines (PE) were negatively associated with Bacteroidales (order level) and positively associated with *Erysipelotrichaceae*.

## Discussion

The current study is a short-term study following opioid administration and analysis of fecal samples collected from mice over 6 days. We demonstrated significant changes in the microbial community within one day post-morphine administration and that morphine treatment results in distinct characteristic microbial and metabolomic signatures that are associated with dysbiosis, featuring increased expansion of potential pathogenic bacterial communities (Figs 1, 2 and 3). Previous studies in our laboratory and other research groups have demonstrated that morphine exposure resulted in an increased risk of virulent bacterial infection, bacterial translocation and lethal gut-derived sepsis^14,33,42,43^. Our current results indicate that the increased abundance of potential pathogenic bacteria following morphine treatment may account for morphine-induced bacterial translocation and sepsis.

Richness of microbial diversity is an indicator of microbial homeostasis and reduced microbial diversity is associated with microbial dysbiosis^44^. Phylogenetic analysis revealed that morphine treatment results in decreased alpha diversity (Fig. 3A,B) and distinct clustering in a beta diversity plot compared to that following placebo treatment (Fig. 3C,D). Morphine treatment is associated with a significant decrease in gut microbial alpha diversity, indicating microbial dysbiosis and increased risk for intestinal infections.

In this study, we reveal that *E. faecalis* expansion is associated with morphine-induced gut dysbiosis. This observation is in alignment with our previous studies where we demonstrate that the major serotype of the bacterial species that translocated into mesenteric lymph nodes and liver following morphine treatment belonged to the *Enterococcus* family^15^. Infection of WT morphine-treated animals with *E. faecalis* may result in greater gut pathology and sustained inflammation (unpublished observation). Transfer of microbial content from morphine-treated animals to antibiotic-treated animals recapitulated morphine-induced pathology^18^. These observations are consistent with *E. faecalis* being associated with ulcerative colitis in humans and with opioid-induced sepsis in a murine model^15,45^. Moreover, we demonstrated that *E. faecalis* augments tolerance of morphine analgesic effect in mice (Fig. 5). These results indicate that expansion of *E. faecalis* may act as biomarker for morphine-induced gut microbial dysbiosis.

This study revealed that morphine-induced changes in the gut metabolomic profile shifts gradually with differential changes in individual metabolites. Differential changes in the gut metabolomics profile may reflect the alteration of the gut microbiome and, therefore, contribute to host response following morphine treatment. Cross-correlation between the gut microbiome and the metabolome indicates an association between bacterial communities and functional metabolites (Supplementary Figure S1). Cholic acid is negatively associated with *Enterococcus* and *Erysipelotrichaceae* but positively associated with *Bacteroidales*. In contrast, PEs and stearic acid are positively associated with *Enterococcus* and *Erysipelotrichaceae* but negatively associated with *Bacteroidales*. Our previous study determined that morphine treatment leads to intestinal barrier dysfunction. It is well-demonstrated that bacterial metabolites regulate GI barrier function by the xenobiotic sensor PXR-dependent TLR4 signaling pathway, suggesting that the alteration of the gut microbiome and metabolome may contribute to gut barrier dysfunction following morphine treatment. Furthermore, bile acids have been demonstrated to mediate host resistance to *C. difficile* infection. Decrease in primary and secondary bile acids in the gut following morphine treatment is associated with an increase in pathogenic gut bacteria, such as *E. faecalis*. This is consistent with observations that demonstrate that switching to a high-fat diet in mice is characterized with *Erysipelotrichaceae* increase and associated with increased risk of infectious diseases and inflammation^46,47^.

Gut metabolites are an important link between gut microbes and host biological functions. Morphine treatment results in dramatic changes in the fecal metabolome, alters fatty acid and bile acid metabolism, and increases PEs levels in the gut. PEs are the primary lipid components of the inner bacterial membrane. An increase in PE levels is indicative of significant cell injury. PEs are also associated with bacterial stress responses^48^.

This is the first study examining morphine metabolites in both stool and serum samples in a time-dependent manner. We clearly demonstrate that enterohepatic recirculation of morphine decreases over time, implicating, for the first time, the role of the gut microbiome in modulating morphine pharmacokinetics. We further establish that the bacterial communities that synthesize β-glucuronidase, which is associated with bile metabolism, also play a significant role in morphine metabolism. Key morphine metabolites, M6G and M3G, are hydrolyzed by bacterial β-glucuronidase and are subsequently reabsorbed as morphine. Collectively, these results reveal that opioid-induced alterations of the gut microbiome and metabolome contribute to dysregulation in morphine pharmacokinetics. Our results demonstrate, for the first time, that opioid-induced dysbiosis results in decreased enterohepatic recirculation of morphine into systemic circulation resulting in lower bioavailability of morphine over time, thereby gradually reducing the efficacy of morphine as an analgesic agent. Our studies are consistent with recent studies from Matthew Redinbo’s laboratory at the University of North Carolina at Chapel Hill, demonstrating a significant role of β-glucuronidase enzymes, expressed by the GI microbiota, in mammalian systems and inactivation of endobiotic and xenobiotic compounds^49^.

Morphine metabolism and elimination plays an important role in determining drug pharmacokinetics and assessing drug efficacy and adverse effects in clinical terms. We demonstrated that the M3G/MS serum concentration ratio increased between day 1 and day 6 post-treatment (Fig. 7F), and this ratio also increased in fecal samples between day 1 and day 2 post-morphine treatment, indicating decreased M3G deconjugation within the gut lumen (Fig. 7E). The major glucuronide deconjugating bacteria are strict anaerobes, such as *bacteroides* and *bifidobacteria*, which express β-glucuronidase activity^50^. In the present study, we demonstrate that morphine treatment results in a decrease in *Bacteroidales*, suggesting that the observed decrease in M3G-deconjugation is a consequence of a decrease in deconjugating bacteria (Supplementary Table S1). Our cross-correlation analysis results reveal that these *Bacteroidales* are positively associated with cholic acid and octadecenoic acids, but negatively associated with PEs, glucosides, and stearic acid, which is consistent with what we observed in the morphine-induced alteration of the metabolomic profile (Fig. 6D,F). The phosphatidylethanolamine-binding protein (PEBP) (alternatively named Raf-1 kinase inhibitor protein or RKIP), initially observed to bind PEs, has been demonstrated to be associated with morphine derivatives^51^. PEBP acts as a molecular shield and prevents morphine-3-glucuronide from rapid clearance^52^. These results reveal that the loss of the deconjugating bacterial population, decreases primary and secondary bile acids and modulates morphine metabolism, elimination and enterohepatic recirculation, which consequently impacts the systemic bioavailability of opioids. Therapeutics directed at these targets may prolong the efficacy of opioid use with fewer side effects.

## Conclusion

To our knowledge, this is the first study demonstrating that morphine use/abuse results in the emergence of distinct microbial and metabolomic signatures. Morphine-induced alterations in the gut microbiome and metabolome are inhibited by naltrexone, an opiate receptor antagonist, indicating that morphine-induced changes are opiate receptor-dependent. We further identified *E. faecalis* being strongly correlated with gut dysbiosis following morphine treatment, indicating its potential application in therapeutics and non-invasive diagnostics.

**Figure 8:**
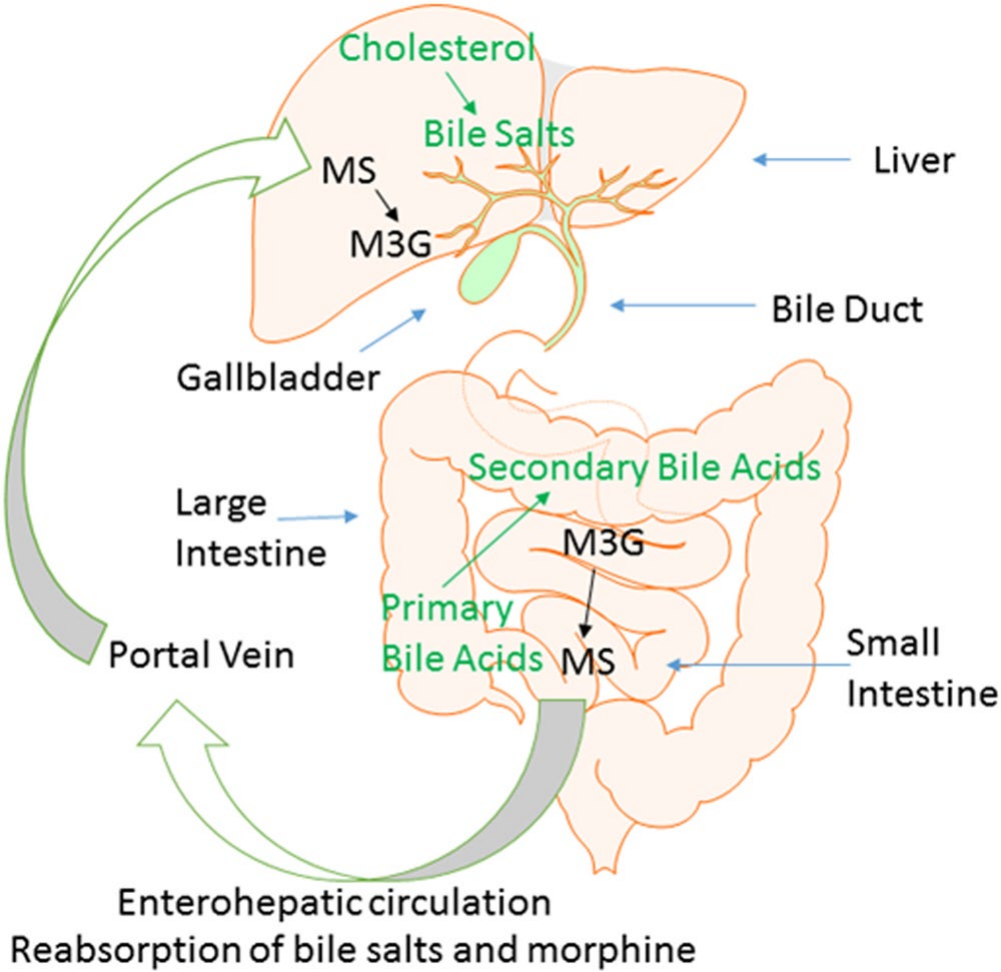
Model of metabolism and biotransformation of morphine and bile acids. In liver, cholesterol is transformed to primary bile acids, and morphine is conjugated to M3G. In the gut, intestinal bacteria transform primary bile acids and M3G into secondary bile acids and morphine, respectively. Bile acids and morphine are reabsorbed and recycled through enterohepatic circulation.

Morphine undergoes rapid first pass metabolism and is metabolized in the liver to M3G and M6G and recirculated back through enterohepatic recirculation (Fig. 8). We investigated the role of the intestinal microbiota on the biotransformation of opioids and other small molecular metabolites. Morphine-induced dysbiosis disrupts morphine metabolism and its enterohepatic recirculation (Fig. 8). Morphine metabolism and elimination plays an important role in determining drug pharmacokinetics and in assessing drug efficacy and adverse effects in clinical terms. By understanding and altering the intestinal microbiota one may be able to detect and minimize the adverse effects of xenobiotics. Thus, intestinal microbiota represent a therapeutic target for prolonging the efficacy of morphine in pain management.

In summary, we demonstrate for the first time that morphine administration results in distinct gut microbial and metabolic changes which impacts morphine metabolism and its pharmacokinetic profile contributing to the deleterious effects of short-term opioid use. Our study implicates that modulation of the gut microbiome may be a plausible therapeutic target in minimizing the negative consequences and prolonging the analgesic function of morphine.

## Methods

### Experimental animals

Pathogen-free C57BL/6J mice were obtained from the Jackson Laboratory (Bar Harbor, Maine, USA). All mice were female and 8–10 weeks old. A maximum of four mice were housed per cage. Food and tap water were available ad libitum. The animal housing facilities were maintained on a 12-h light/dark cycle, with constant temperature (72 ± 1 °F) and 50% humidity. All animals were maintained in specific-pathogen-free facilities and all procedures were approved by the University of Minnesota Institutional Animal Care and Use Committee (IACUC). The IACUC protocol number was 1203A11091. All procedures were conducted in line with the guidelines set forth by the National Institutes of Health Guide for the Care and Use of Laboratory Animals.

### Animal treatment

Mice received morphine through the pellet implantation method, as previously described^53^. Using this method, plasma levels of morphine were maintained in the 0.6–2.0 µg/ml range (range observed in opioid abusers and patients on opioids for moderate to severe pain). Furthermore, this model is commonly used in the study of opiate dependence and addiction^53^. Briefly, placebo or 25 mg morphine or 30 mg naltrexone pellets (National Institutes of Health [NIH]/National Institute on Drug Abuse [NIDA], Bethesda, MD) were inserted in a small pocket created by a small skin incision on the dorsal side of animals; incisions were closed using surgical wound clips (Stoelting, 9 mm Stainless Steel, Wooddale, IL).

### Experiment of determining effects of *E. faecalis* on morphine tolerance

The mice were administrated with 5 g/L of streptomycin sulphate in the drinking water for 2 days and switch to normal drinking water for 24 hours before *E. faecalis* inoculation by oral gavage. The spectinomycin sulphate selective *E. faecalis* were diluted up to the concentration of 2 × 10^10^/mL in PBS. Each mouse was administered with 200 ul spectinomycin solution by oral gavage daily. After 48 hours post gavage, the mice were treated with 250 mg/L spectinomycin sulfate in the drinking water during the behavior study. To maintain the population of *E. faecalis* in the mouse gut, mice were administered with the same dose of *E. faecalis* and the same dose of spectinomycin sulphate by oral gavage daily during the behavior experiment for 8 days.

Analgesic effectiveness of morphine interpreted as MPE% (percentage of maximum possible effect), was determined by tail flick analgesic test^54^. Mice were intraperitoneally injected with saline or 15 mg/kg morphine twice daily for 8 days with 12 hours interval. Behavioral assessment was performed before and 30 min after saline or morphine administration in the morning. Placebo + PBS group and Placebo + *E. faecalis* group served as control groups. All groups had a minimum of 10 mice per group. New groups of mice in the Placebo + PBS and Placebo + *E. faecalis* were administered with 15 mg/kg morphine at each time point to test analgesic reaction of these naive mice to morphine treatment. Withdrawal latencies of the tail from a radiant heat source were measured by tail flick. Voltage to the light source was adjusted to achieve baseline latency between 2–3 seconds. The cut-off time is 10 seconds to avoid tissue damage. The mice were placed on the tail flick assay for 5 min for habituation everyday for two days before the behavior test. Everyday, the averages of each two measurements before and after morphine injection were recorded as baseline and then response to morphine antinocipective effect were recorded. The tests of significance were performed using a two-sided student’s two-sample t-test. P value of 0.05 or less was considered significant.

### Fecal sample collection and DNA extraction

Stool samples were collected in 1.7 ml RNase/DNase-free tubes (Catalog #: C-2170, Denville Scientific, Holliston, MA, USA) at various time points. The fecal samples were immediately frozen on dry ice and then stored at −80 °C; DNA extractions from the fecal matter was carried out using the PowerSoil DNA isolation kit (Catalog #: 12888–100, MO BIO Laboratories, Carlsbad, CA, USA). All extracted DNA samples were stored at −80 °C until amplification.

### Quantitative real-time PCR amplification for Illumina sequencing

The 16S sequencing procedure was performed at the University of Minnesota Genomic Center. Method optimizations and the protocol have been recently published^55,56^. Fecal DNA samples (25 ng) were used as templates for PCR amplification of the V4 region of the 16S rRNA gene. Degenerate primer sets were designed with Illumina index sequences on the 5′ end of the reverse primer, which were specific to each fecal DNA sample and allowed for multiplex sequencing. Primers also contained Illumina PCR primer sequences (reverse primer) and Illumina TruSeq Universal Adapter sequences (forward primers) for library creation. The primer sequences (16S-specific portion in bold) used were Meta_V4_515F (TCGTCGGCAGCGTCAGATGTGTATAAGAGACAG**GTGCCAGCMGCCGCGGTAA**) and Meta_V4_806R (GTCTCGTGGGCTCGGAGATGTGTATAAGAGACAG**GGACTACHVGGGTWTCTAAT**). The indexing primers are as follows: This step adds both the index and the flow cell adapters. [i5] and [i7] refer to the index sequence codes used by Illumina. The p5 and p7 flow cell adapters are in bold. Forward indexing primer: **ATGATACGGCGACCACCGA**GATCTACAC[i5]TCGTCGGCAGCGTC; Reverse indexing primer: **CAAGCAGAAGACGGCATACGA**GAT[i7]GTCTCGTGGGCTCGG. PCR reactions were performed using KAPA HiFidelity Hot Start Polymerase. PCR 1 (using the Meta_V4_515F/Meta_V4_806R primer pair): 95 °C 5 minutes, 20 cycles (98 °C 20 seconds, 55 °C 15 seconds, 72 °C 1 minute), followed by holding at 4 °C. After the first round of amplification, PCR 1 products were diluted 1:100 and 5 µl of 1:100 PCR 1 was used in the second PCR reaction. PCR 2 (using different combinations of forward and reverse indexing primers): 95 °C 5 minutes, 10 cycles (98 °C 20 seconds, 55 °C 15 seconds, 72 °C 1 minute), followed by holding at 4 °C.

### DNA sequencing

Genomic DNA sequencing was performed using Illumina MiSeq at the University of Minnesota Genomic Center (UMGC). Pooled, size-selected samples were denatured with NaOH, diluted to 8 pM in Illumina’s HT1 buffer, spiked with 15% PhiX, and heat-denatured at 96 °C for 2 minutes immediately prior to loading. The MiSeq 600 cycle v3 kit was used to sequence the sample. Nextera adapter sequences for post-run trimming were as follows:

Read 1: CTGTCTCTTATACACATCTCCGAGCCCACGAGACNNNNNNNNATCTCGTATGCCGTCTTCTGCTTG

Read 2: CTGTCTCTTATACACATCTGACGCTGCCGACGANNNNNNNNGTGTAGATCTCGGTGGTCGCCGTATCATT.

The raw data files for 16s rDNA sequencing have been deposited with ArrayExpress with the accession numbers E-MTAB-5546 and E-MTAB-5550.

### Sequence processing and analysis

Microbial operational taxonomic units (OTUs) and their taxonomic assignments were obtained using default settings in QIIME version 1.8.0 by reference-mapping at 97% similarity against representative sequences of 97% OTU in Greengenes (release GG_13_8), following which chimeric sequences were removed from subsequent analyses^57^. Sequences showing 97% or greater similarity were clustered into operational taxonomic units (OTUs) using the USEARCH method and representative sequences were assigned taxonomies using the RDP classifier.

### Quantitative real-time PCR amplification for detection of specific species

Fecal DNA samples (25 ng) were used as templates for PCR amplification of the species-specific primers. Amplification of species-specific primers was normalized to the 16S rRNA gene. 16S rRNA gene universal primers used were as follows: F: ACTCCTACGGGCAGCAG; R: ATTACCGCGGCTGCTGG. *Enterococcus faecalis*, F: ATCAAGTACAGTTAGTCTT, R: ACGATTCAAAGCTAACTG; *Enterococcus faecium*, F: GCAAGGCTTCTTAGAGA, R: CATCGTGTAAGCTAACTTC; *Fusobacterium nucleatum*, F: AGA GTT TGA TCC TGG CTC AG, R: GTC ATC GTG CAC ACA GAA TTG CTG; fadA, F: CAC AAG CTG ACG CTG CTA GA, R: TTA CCA GCT CTT AAA GCT TG; *Clostridium difficile*, F: TTG AGC GAT TTA CTT CGG TAA AGA, R: CCA TCC TGT ACT GGC TCA CCT; *Clostridium scindens*, F: CGT AAC GCG CTC TTT CTT CG, R: CCT TCC TCC AGG TTC TCC CT; *Coprobacillus catenaformis*, F: CGGACGCGATGCTTCT(A/G)GC, R: AACATATCTCCCATGCGGTTG; *Staphylococcus aureus*, F: GCGATTGATGGTGATACGGTT, R: AGCCAAGCCTTGACGAACTAAAGC; the qPCR program was as follows: (i) initial denaturation at 95 °C (10 min) and (ii) 45 cycles of 95 °C (15 sec), 60 °C (45 sec followed by fluorescence plate read).

### LC-MS analysis of fecal extracts

The method for LC-MS analysis of fecal extracts was as described in a previous study with slight modification^58^. Fecal samples were suspended in 1 ml of 50% acetonitrile (wt/vol) and extracted by vortexing and sonication for 10 min. The suspension was extracted twice by collecting supernatant after centrifuging at 18,000 × *g* for 10 min. After passage of the supernatant through a 2-μm filter, the filtrate was transferred to a UPLC vial and subjected to LC-MS analysis. A 5-μl aliquot prepared from the fecal extract was injected into an Acquity ultra-performance liquid chromatography (UPLC) system (Waters, Milford, MA) and separated in a BEH C18 column (Waters). The mobile phase used a gradient ranging from water to 95% aqueous ACN containing 0.1% formic acid over a 10-min run. The LC eluent was introduced into a Xevo-G2-S quadrupole time-of-flight mass spectrometer (QTOFMS, Waters) for accurate mass measurement and ion counting in negative-mode. Capillary voltage and cone voltage for electrospray ionization was maintained at −3 kV and −35 V for negative-mode detection. Source temperature and desolvation temperature were set at 120 °C and 350 °C, respectively. Nitrogen was used as both cone gas (50 L/h) and desolvation gas (600 L/h), and argon as collision gas. For accurate mass measurement, the mass spectrometer was calibrated with sodium formate solution with mass-to-charge ratio (*m/z*) of 50–1,000 and monitored by the intermittent injection of the lock mass leucine enkephalin ([M-H]^−^ = *m/z* 554.2615) in real time. Mass chromatograms and mass spectral data were acquired and processed by the MassLynx^TM^ software (Waters) in centroided format. Additional structural information was obtained by tandem MS (MS/MS) fragmentation with collision energies ranging from 15 to 40 eV. The concentrations of bile acids in fecal samples were determined by calculating the ratio between the peak area of individual bile acids and the peak area of internal standard and then fitting with a standard curve using the QuanLynx^TM^ software (Waters). Morphine sulfate standard was purchased from National Institute of Drug Abuse (NIDA) and M3G standard was purchased from Sigma.

### Statistics

For *microbiome analysis*, QIIME 1.8 was used to calculate the α diversity (alpha_rarefaction.py) and to summarize taxa (summarize_taxa_through_plots.py). Principal coordinates analysis (PCoA, = multidimensional scaling, MDS) is a method to explore and to visualize inter-object similarity/dissimilarity in a low-dimensional, Euclidean space. Principal coordinate analysis (PCoA) of unweighted UniFrac phylogenetic distances between microbial communities were carried out using this program with observation ID level. The input for PCoA is the OTU table containing the number of sequences observed in each OTU (rows) for each sample (columns). OTU tables were rarefied to the sample containing the lowest number of sequences in each analysis. An estimate of the false discovery rate (q-value < 0.10) was calculated to take into account the multiple comparisons that occur in microbiome analysis. P-value in UniFrac distance comparison was Bonferroni-corrected. The tests of significance were performed using a two-sided student’s two-sample t-test.

For *real-time PCR analysis*, the heat map was generated using a log transformation of the real-time PCR data presented as ΔCT (CT_species – CT_universal_16 SrRNA). Red color indicates increased levels of amplification. The 16S-rRNA gene amplification fold change due to treatments were analyzed by the t-test or ANOVA (GraphPad Prism).

For *metabolite analysis*, experimental values for fecal metabolites are expressed as the means ± SEM. Scores scatter plots of the partial least square discriminant analysis (PLS-DA) model were constructed. An estimate of the false discovery rate (q-value < 0.10) was calculated to take into account the multiple comparisons that normally occur in metabolomic-based studies. In this study, the microbiome and metabolic profiles of fecal samples from 4 groups (placebo, morphine, morphine + naltrexone, naltrexone) were compared. For morphine and M3G concentration analysis, statistical analysis was performed using t-test or ANOVA (GraphPad Prism).

*Cross-correlation analysis* between the gut microbiome and metabolome data was performed using the Microbiome R package, following the instructions provided online^59^. Cross-correlation between phylogenotypes of microbiome and metabolites was analyzed using Spearman correlation (Supplemental Fig. 1). Taxa relative values were transformed as the base-10 logarithm of a number before being correlated with metabolomic relative abundance values and FDR-adjusted p-value (q-value) was obtained. The URL of the Microbiome R package is: http://microbiome.github.com.

### Data availability

The microbiome datasets supporting the conclusions of this article are available in the ArrayExpress repository. The accession numbers are E-MTAB-5546 and E-MTAB-5550. The hyperlink to datasets is https://www.ebi.ac.uk/arrayexpress/.

## Electronic supplementary material

Supplementary Figure S1

Supplementary Table S1
