# Supplementary material for: Morphine induces changes in the gut microbiome and metabolome in a morphine dependence model
Source: Sci Rep. 2018 Feb 26;8:3596. doi: 10.1038/s41598-018-21915-8 (PMC5827657; doi:10.1038/s41598-018-21915-8)
Supplement: Supplementary file 1 — Supplementary Figure S1 [file 41598_2018_21915_MOESM1_ESM.pdf]

Title

Morphine induces changes in the gut microbiome and metabolome in a morphine dependence model

Fuyuan Wang[1], Jingjing Meng[2], Li Zhang[3], Timothy Johnson[4], Chi Chen[5], Sabita Roy[1,2,3]

Department of Veterinary Population Medicine[1]

Department of Pharmacology[3]

Department of Veterinary and Biomedical Sciences[4]

Department of Food Science and Nutrition[5]

515 Delaware St SE, Moos 11-204, University of Minnesota, Minneapolis, MN 55455, USA

Department of Surgery and Sylvester Cancer Center, Miller School of Medicine, University of Miami, Miami, Florida 33101, USA[2]

Correspondence

Sabita Roy, Ph.D., Professor and Associate Vice Chair for Career Development, Department of Surgery, Miller School of Medicine, University of Miami, Miami, Florida 33101

Tel: 305-243-8452

E-mail address: sabita.roy@miami.edu

Supplementary Figure S1

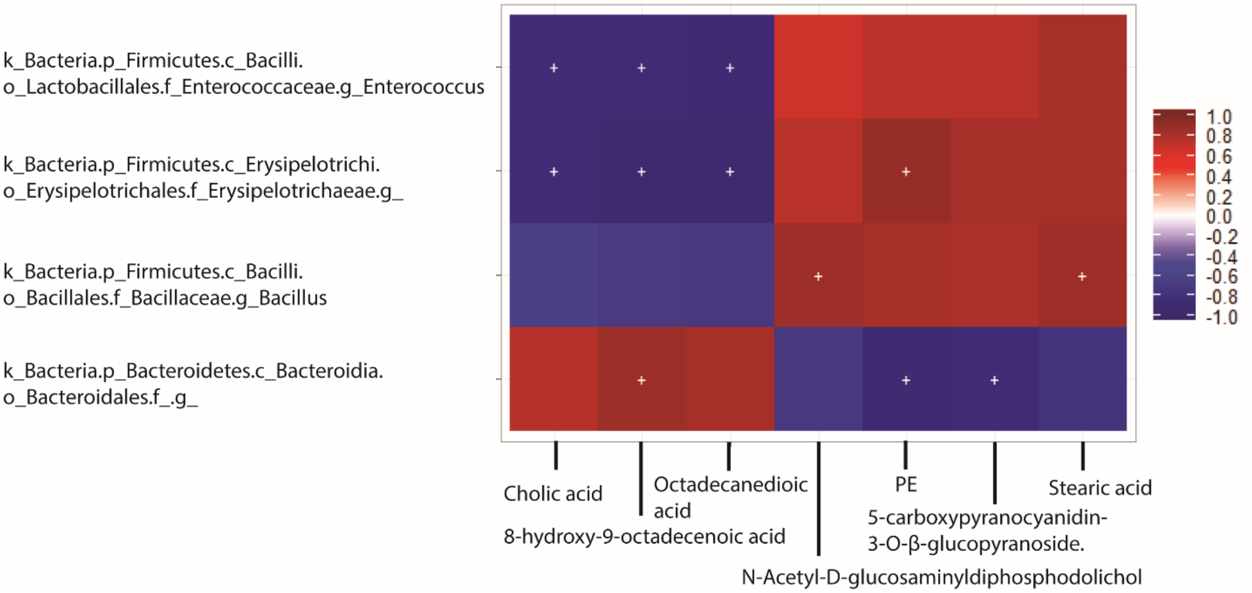

Supplemental Figure S1. Cross-correlation analysis between the gut microbiome and metabolome. Cross-correlation between phylogenotypes of the microbiome and metabolites was analyzed using Spearman correlation. Taxa relative values were transformed as the base-10 logarithm of a number before being correlated with metabolomic relative abundance values. The test of significance was performed using the multiple hypothesis testing method. A corrected p-value (the false discovery rate corrected) was also computed for each test.
